# Decoding functional impact of epigenetic regulator mutations on ligand–receptor interaction perturbations for evaluation of cancer immunotherapy

**DOI:** 10.1111/jcmm.70009

**Published:** 2024-09-25

**Authors:** Aiai Shi, Chaohuan Lin, Jie Lyu

**Affiliations:** ^1^ Joint Centre of Translational Medicine, Wenzhou Institute University of Chinese Academy of Sciences Wenzhou Zhejiang People's Republic of China; ^2^ Institute of Theoretical Physics Chinese Academy of Sciences Beijing People's Republic of China; ^3^ Joint Centre of Translational Medicine the First Affiliated Hospital of Wenzhou Medical University Wenzhou Zhejiang People's Republic of China; ^4^ Postgraduate Training Base Alliance of Wenzhou Medical University Wenzhou Zhejiang People's Republic of China; ^5^ Oujiang Laboratory (Zhejiang Lab for Regenerative Medicine, Vision and Brain Health) Wenzhou Zhejiang People's Republic of China

**Keywords:** cancer, epigenetic regulator, immune, immunotherapy, ligand–receptor interaction, mutation

## Abstract

Cellular crosstalk mediated by ligand–receptor interactions largely complicates the tumour ecosystem, resulting in heterogeneous tumour microenvironments that affect immune response and clinical benefits from immunotherapy. Epigenetic mechanisms are pivotal to expression changes of immune‐related genes and can modulate the anti‐tumour immune response. However, the functional consequences of disrupted epigenetic regulators (ERs) on ligand–receptor interactions in the tumour microenvironment remain largely unexplored. Here, we proposed mutations of ERs in perturbed interactions (MERIN), a molecular network‐based approach that incorporates multi‐omics data, to infer the potential consequences of ER mutations on ligand–receptor interaction perturbations. Leveraging cancer genomic profiles and molecular interaction data, we comprehensively decoded the functional consequences of ER mutations on dysregulated ligand–receptor interactions across 33 cancers. The dysregulated ligand–receptor genes were indeed enriched in cancer and immune‐related function. We demonstrated the potential significance of PD1–PDL1 interaction‐related ER mutations in stratifying cancer patients from multiple independent data cohorts. The ER mutation group showed distinct immunological characterizations and prognoses. Furthermore, we highlighted that the ER mutations could potentially predict clinical outcomes of immunotherapy. Our computational and clinical assessment underscore the utility of MERIN for elucidating the functional relevance of ER mutations in cancer immune response, potentially aiding patients' stratification for immunotherapy.

## 1 INTRODUCTION

The complexity of tumours is reflected by complicated and dynamic crosstalk among microenvironment cells and molecules.^1^ Cellular crosstalk mediated by ligand–receptor interactions in the tumour microenvironment (TME) plays a crucial role in the processes of tumorigenesis, progression and therapy resistance.^1^, ^2^ For example, the ligand–receptor interaction of PDL1–PD1 can mediate the crosstalk between tumour cells and CD8 T cells, thereby suppressing anti‐tumour immunity by delivering negative signalling to CD8 T cells.^3^ The utilization of immune checkpoint blockade (ICB) to target cell–cell crosstalk has significantly advanced clinical cancer treatment, particularly through the inhibition of PD1 or CTLA4‐related ligand–receptor interactions.^4^ However, tumour cells have developed mechanisms to evade immune surveillance by manipulating molecular interactions that impact the effector function of immune cells,^5^, ^6^ leading to restricted clinical efficacy. Thus, a better understanding of the molecular mechanisms behind the dysregulated ligand–receptor interactions is of great importance.

Studies have revealed the role of epigenetic mechanisms in the dysregulated anti‐tumour immune response.^7^, ^8^ Epigenetic regulation plays a crucial role in altering the expression of immune checkpoints, immune infiltrates and other immune‐related pathways, leading to immune evasion.^9^ Consistently, Anders et al. reported the differential epigenetic modifications of immune checkpoint and costimulatory genes that mediate the tumour‐immune crosstalk in cancer, affecting effector T cell recruitment to the TME and thereby cancer prognosis.^8^ Epigenetic regulators (ERs) are important upstream regulatory machinery of gene expression,^10^, ^11^ and their dysfunction by genetic alterations could induce gene expression changes in cancer, contributing to tumorigenesis.^12^, ^13^ Recent research studies have shed light on the importance of dysfunctional ERs in modulating the immune response, consequently affecting immunotherapy efficacy.^14^ For example, the mutations in tumour protein p53 (TP53) gene could modulate the recruitment and activity of immune cells in cancer, leading to suppressive immune response.^15^ Mutations in histone–lysine N‐methyltransferase 2 (KMT2) family genes were associated with boosted tumour immunogenicity and better immunotherapy response.^16^ Inhibition of histone deacetylase 1 (HDAC1) could sensitize tumour cells to T‐cell‐mediated immune response^17^ and stimulate PDL1 expression for enhanced immunotherapy response in cancers.^18^, ^19^ However, there is a lack of comprehensive decoding of the functional impact of ER mutations on dysregulated ligand–receptor interactions in the TME, impeding the elucidation of key regulatory molecules involved in dysregulated immune microenvironment.

In this study, we developed a network‐based computational approach mutations of ERs in perturbed interactions (MERIN) to infer the functional consequences of ER mutations on dysregulation of immune‐related molecular interactions. Leveraging genomic profiles and ligand–receptor interaction pairs, we applied the approach to 33 cancer types to elucidate the associations between ER mutations and dysregulated ligand–receptor interactions. Moreover, we constructed an ER mutation‐based signature as the potential predictor of cancer immunotherapy response in both different cancers and data cohorts.

## 2 METHODS

### 2.1 Data collection of molecular profiles

The genome‐wide molecular profiles of more than 10,000 cancer patients from 33 cancer types were obtained from the TCGA project. The cancer types and the corresponding number of tumour samples are available in Table S1. For the gene expression data of these patients, we used the transcripts per million values^20^ and retained genes with expression in more than 70% tumour samples. For the somatic mutation data, we downloaded the public Mutation Annotation Format file (https://gdc.cancer.gov/about‐data/publications/pancanatlas) and retained mutations that affect protein‐coding capacity. Mutation profiles were then represented as binary matrixes, where ‘1’ indicates mutations of a specific gene occurred in one tumour sample, and ‘0’ indicates wild‐type status. Clinical data of these tumour samples was also downloaded to perform survival analysis (https://gdc.cancer.gov/about‐data/publications/pancanatlas). We also obtained public genomic profiles and clinical information for cancer samples treated with ICB from cBioPortal (https://www.cbioportal.org).^21^

We acquired values of the immune‐related features characterizing TCGA tumour samples from Thorsson et al.,^22^ including some key immune function and immune cell infiltration calculated from CIBERSORT. We measured the values of MHC‐I expression and cytotoxic T lymphocyte (CTL) through calculating the arithmetic mean of genes expression in the tumour samples.^23^ Genes HLA‐A, HLA‐B, HLA‐C, B2M were used for major histocompatibility complex class I (MHC‐I) expression, and CD8A, CD8B, GZMA, GZMB, PRF1 were used for CTL level. The cytolytic activity (CYT) was measured from the geometric mean of GZMA and PRF1 expression.^24^

### 2.2 Collection of epigenetic regulators and ligand–receptor interaction pairs

We compiled a total of 690 human ER genes annotated with available ‘histone_type’ or ‘Methylator_type’ information from a published study (Table S2).^25^ The ER gene list and the class annotations (e.g. histone reader) used in our study are available in Table S2. We obtained the human ligand–receptor interactions used in the tumour immune microenvironment study for our analysis.^3^ These interactions were integrated from five known sources and evidenced by at least two sources, and a total of 3182 interaction pairs were finally obtained. The gene names of ligand–receptor pairs (e.g. PDL1–PD1) for the subsequent analysis are listed in Table S3.

### 2.3 MERIN: Identifying ER mutation‐mediated ligand–receptor interaction dysregulation

**FIGURE 1:**
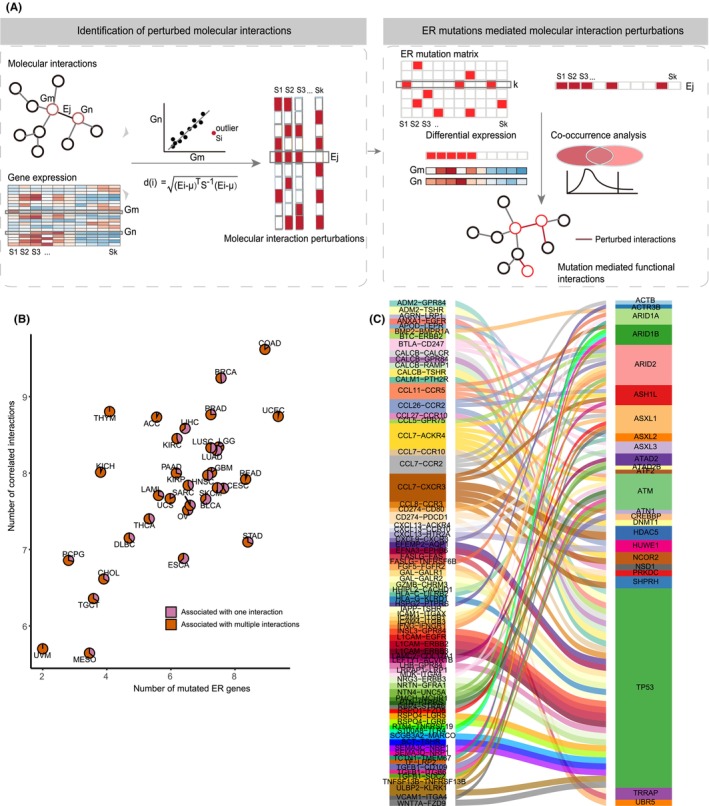
Detection of ER mutations correlated with ligand–receptor interactions in cancer. (A) Overview of the MERIN approach for decoding ER mutation effect on molecular interactions. (B) The distribution of identified ER mutations (x‐axis) and ligand–receptor pairs (y‐axis) across 33 cancer types. The number is log‐transformed. Pie charts show the proportion of ER mutations related to one (purple) or more (orange) ligand–receptor interactions. (C) Top 100 ER–interaction association pairs by occurred cancers. The number regarding occurred cancers is represented by the height of a stream field.

To identify the potential ER mutations involved in dysregulated molecular interactions in cancer, we designed a network‐based integrative method (Figure 1A) referring to the previous network‐based correlation analysis method of genomic mutations.^26^ Four types of molecular data were required by MERIN as the inputs: a somatic mutation matrix representing the gene mutation status (‘1’ or ‘0’) in each tumour sample, a gene expression matrix of tumour samples, predetermined ligand–receptor interaction pairs and ER gene set. The method primarily consists of two steps: (i) identification of dysregulated ligand–receptor interaction pairs and (ii) selection of ER mutations involved in interaction dysregulation.

Given the ligand–receptor interaction network, we identified the dysregulated molecular interaction pairs in each sample based on their expression correlation. We considered that one specific interaction is dysregulated in a sample only if the distance of this sample to the other samples is larger than expected in the two‐dimensional space of one interaction pair. For each ligand–receptor pair (*G*_*i*_, *G*_*j*_), we defined the two‐dimensional gene expression vector in sample *m* as *E*_*m*_ = (*e*_*im*_, *e*_*jm*_). First, we calculated the mahalanobis distance *d*_*m*_ of sample *m* and sample mean *μ*:

(1) $$d\left(m\right)=\sqrt{{\left(E_{m}-\mu \right)}^{T}S^{-1}\left(E_{m}-\mu \right)}$$

where *μ* and *S* represent the mean value and covariance of gene expression in all samples, respectively. We calculated the distance for all samples based on Equation (1). Then, we detected the outliers based on the overall distribution of these calculated distances using the Grubbs' test. We measured the outliers with the value *G*, which was calculated by the distance *d*_*m*_ of sample *m*, mean $\bar{d}$ and standard deviation *s* of all samples (Equation (2)). The samples with statistically significant *G* values were all determined as showing the ligand–receptor interaction dysregulation.

(2) $$G=\frac{\max\left(d_{m}-\bar{d}\right)}{s}$$

After detecting the dysregulated samples for all ligand–receptor pairs, we then selected potential ER mutations that mediated the interaction dysregulation by integrating the ER gene mutation and ligand–receptor dysregulation profiles of all tumours. For each ER gene, we examined the expression changes of the ligand–receptor interacting genes and assessed the degree to which mutations in the ER gene and dysregulated interactions co‐occurred. In brief, we required that at least one interacting gene of a dysregulated interaction was differentially expressed in the mutation samples. This was achieved using Wilcoxon rank‐sum test (*p* < 0.05 as significant). Next, we assessed the statistical significance of sample overlap between the ER mutated and interaction dysregulated tumour samples using a permutation test. We considered that more samples they were overlapped, more likely the interaction was dysregulated by the ER mutation. The mutation samples were permuted 1000 times to generate a background distribution which was used to evaluate the statistical significance of an observed overlap (Equation (3)). We selected ER mutations with *p* < 0.05 as the potential mediators of ligand–receptor interaction dysregulation.

(3) $$p=\frac{\#\left(n_{\text{permu}}>n_{\text{obser}}\right)}{N}$$

where *n*_permu_ and *n*_obser_ represent the number of samples with both the ER mutation and the specific dysregulated interaction in permuted and observed conditions, respectively. *N* represents the number of permutations.

### 2.4 Functional analysis of the ER mutation‐mediated ligand–receptor interactions

To assess functional significance of dysregulated ligand–receptor genes in cancer, we performed the functional enrichment analysis using 50 known cancer‐associated gene sets from the ‘hallmark gene sets’ collection of Molecular Signature Database (MSigDB) via clusterProfiler (version 4.10.0) package. For the identified ER mutations and dysregulated ligand–receptor pairs across all cancer types, we mapped them on the protein–protein interaction (PPI) network consisting of ~19,700 proteins obtained from the STRING database (version 12.0). We focused only on the high‐confidence interactions (i.e. interactions with a score larger than 700). Densely connected modules were identified through the use of Molecular Complex Detection (MCODE) algorithm^27^ and we used Cytoscape to visualize the interaction network.

### 2.5 Construction of the ER mutation signature

To explore the clinical significance of ER mutations related to PD1–PDL1 interaction dysregulation, we stratified tumour samples into different groups by the integrated mutation status of these ER genes: samples with no mutations in any of the ER genes (‘0’: WT group), samples with mutations in only one ER gene (‘1’: single‐mutation group), and samples with two or more ER gene mutations (‘2’: multiple‐mutation group).

### 2.6 Statistical analysis

We achieved the comparisons between two patient groups by two‐sided Wilcoxon rank‐sum method. The Kruskal–Wallis test was used to determine the significance among three patient groups. We assessed Kaplan–Meier survival curves via log‐rank method. The hazard ratios (HRs) with corresponding *p* values were measured via Cox proportional hazard regression model. We achieved the survival analysis via survival (version 3.5.0) and survminer (version 0.4.9) packages. All analyses were conducted by R 4.2.0.

## 3 RESULTS

### 3.1 Identification of ER mutations involved in perturbed ligand–receptor interactions across cancers

To identify potential mutations contributed to the dysregulated molecular interactions in cancer, we assumed that the molecule‐molecule relationships would be perturbed in samples also showing ER mutations. Here, we proposed MERIN utilizing the data of gene expression, somatic mutations and ligand–receptor pairs (Figure 1A). Briefly, we first identified tumour sample‐specific interaction perturbations by examining expression distribution of two interacting genes across all samples on a two‐dimensional space. Then, we assessed the expression changes of these interacting genes in samples with specific ER mutations. Finally, we quantified the extent to which the ER mutations occurred in the samples with a perturbed interaction by a simulation‐based approach.

To explore the landscape of ER mutation‐mediated ligand–receptor dysregulation in cancer, we applied MERIN to 33 TCGA cancers including >10,000 tumour samples (Table S1). A total of 690 ERs annotated by functional types (e.g. histone writer, Table S2) were used in this work.^25^ Then, 3182 known ligand–receptor interactions annotated by the published studies were obtained^3^ (Table S3). On average, we found 132 ER mutations and 264 dysregulated ligand–receptor interactions in cancer. In the majority of cancers, more than 50% of the ER mutations were involved in different interactions (Figure 1B). We focused on the top 100 ER‐interaction pairs ranked by the cancer numbers in which they occurred (Figure 1C, Table S4), and a total of 25 ERs and 78 interactions were shown. As the most frequently identified ER in 26 cancers (Figure S1A), TP53 mutation was involved in the ERBB signalling and ligand–receptor pairs correlated with chemokines, e.g. L1CAM‐ERBB2 and CCL8‐CCR3 (Figure 1C). TP53 mutation in cancers was reported to influence recruitment and function of immune cells, causing cancer development.^15^ The dysfunction of ERBB‐family genes was related to anti‐tumour immune activity, affecting patients' survival and response to immunotherapy.^28^ Mutation of histone writer PRKDC was associated with the CD274 (encoding PDL1)‐PDCD1 (PD1) dysregulation (Figure 1C). Studies have reported the association between PRKDC mutation and PDL1 expression in cancer, and evidenced the ability of PRKDC to be both the biomarker and drug target for checkpoint blockade therapy.^29^, ^30^

Focusing on specific cancers, we still found significant ERs implicated in tumour immunity. As the top one ERs ranked by their associated interactions in lung adenocarcinoma (LUAD), KEAP1 mutation could affect treatment outcomes of ICB through modulating immune infiltration and immune‐related pathways in lung cancer.^31^ SMARCA4 mutation was also reported to influence ICB treatment for lung cancer.^32^ Similarly, KDM5C mutation identified in skin cutaneous melanoma (SKCM) was associated with immune infiltration and inflamed anti‐tumour immunity, affecting treatment outcomes of ICB in cancer.^33^

### 3.2 Functional characterization of ER mutation‐mediated interactions

**FIGURE 2:**
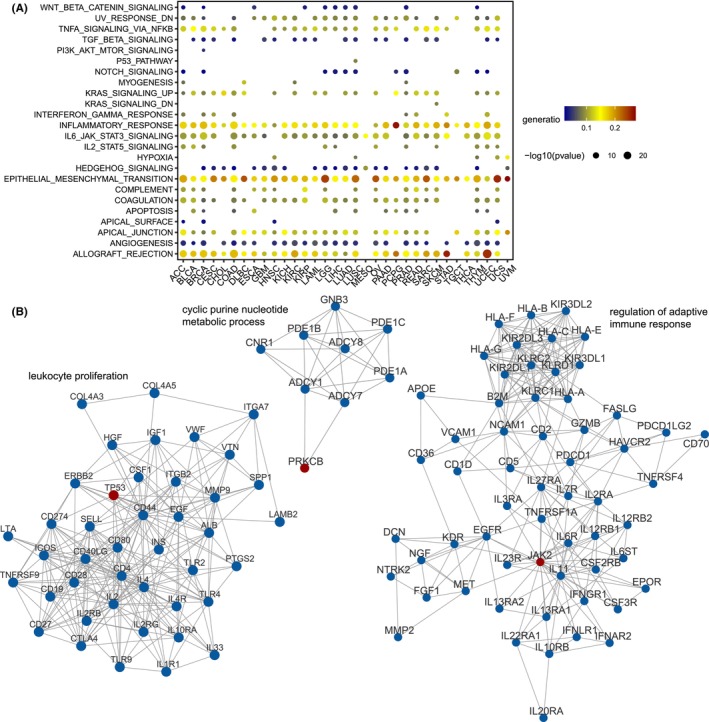
Biological function of ER mutation‐mediated ligand–receptor interactions. (A) Bubble chart showing the enriched hallmark gene sets for interacting ligand–receptor genes. The proportion of overlapped genes upon hallmark sets is indicated by different colours. (B) The densely connected modules identified from protein–protein interaction network by MCODE. The red and blue nodes represent ERs and ligands/receptors, respectively.

To better understand the functional significance of the ER mutation‐mediated ligand–receptor interactions in cancer, we then investigated the genes of these interactions by known cancer gene sets (‘hallmark gene sets’) in Molecular Signature Database (MSigDB).^34^ The ligand–receptor genes were indeed enriched in cancer and immune‐related functions in multiple cancer types (hypergeometric test, false discovery rate <0.05, Figure 2A), such as ‘epithelial_mesenchymal_transition’, ‘allograft_rejection’ and ‘inflammatory_response’. Furthermore, we found that the majority of identified ERs were related to immune infiltration (Figure S2A,B, Table S5). For instance, more than 50% of the ERs were related to CD8 T‐cell infiltration in SKCM and LUAD.

To further characterize the ER mutation‐mediated interaction dysregulation, we explored the subnetwork of these identified ER genes and ligand–receptor genes in the PPI network obtained from STRING database (Figure S2C). By MCODE method,^27^ we found three densely connected modules involving in TP53, JAK2 and PRKCB (Figure 2B). As the dysregulated genes mediated by TP53 in our results (Figure 1C, Table S4), ERBB2 and CD274 were also the neighbouring genes of TP53 in the PPI network, demonstrating the direct association between them (Figure 2B). We then conducted functional enrichment analysis against Gene Ontology biological processes to annotate the biological function of the module genes. These modules were characterized by immune‐related function, such as leukocyte proliferation and regulation of adaptive immune response (Figure 2B, Table S6).

### 3.3 ER mutations involved in the PD1–PDL1 interaction classify distinct cancer patients

**FIGURE 3:**
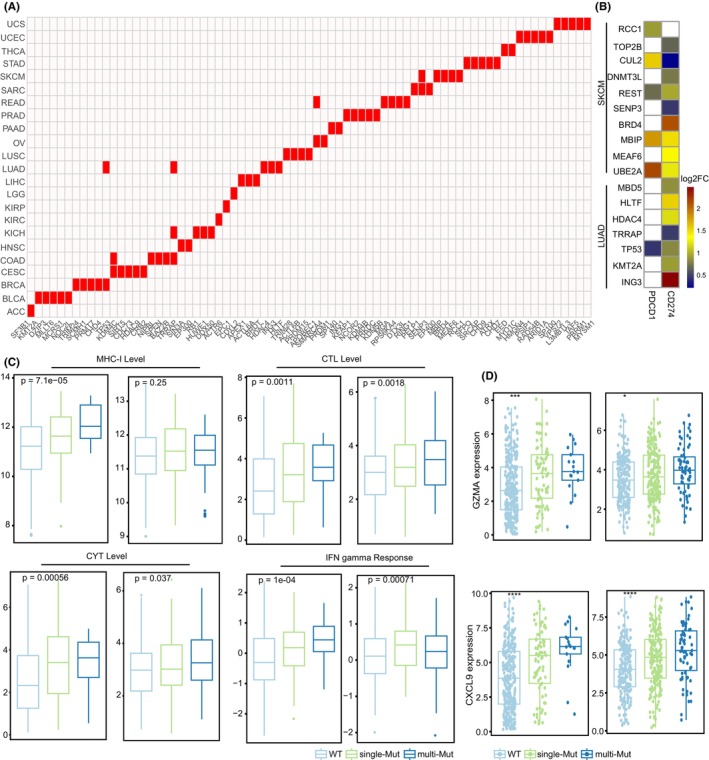
Analysis of ER mutations involved in PDL1‐PD1 interactions in cancer. (A) Top five ER mutations related to PDL1–PD1 interaction dysregulation (ranked by their *p* values) in each of 23 cancer types. (B) Heatmap showing the fold change (log‐transformed) of PDL1/PD1 expression between associated ER mutation and wild type samples in two cancer types of SKCM and LUAD. Significant expression change with *p* < 0.05 are showed. (C) Comparison of immune‐related features among three patient groups stratified by the mutation status of ERs related to dysregulated PDL1–PD1 interaction in SKCM (left) and LUAD (right). (D) Box plots showing the expression distribution of two effector molecules among three patient groups indicated by ‘WT’ (wild type), ‘single‐Mut’ (single‐mutation) and ‘multi‐Mut’ (multiple‐mutation) in SKCM (left) and LUAD (right). ns, non‐significant, *p < 0.05, **p < 0.01, ***p < 0.001 and ****p < 0.0001.

Tumour cells can utilize the immune checkpoint‐related interactions, such as PD1–PDL1 interaction, to induce an immunosuppressive tumour microenvironment. As a result, we found dozens of ER mutations involved in the PD1–PDL1 dysregulation across 23 cancer types (*p* < 0.05, Figure 3A). Indeed, two checkpoint genes were significantly upregulated in ER mutated tumour samples (fold change >1, *p* < 0.05, Figure 3B, Table S7). As in SKCM, PDL1 showed higher expression levels in the BRD4 mutated samples (Figure 3B). Studies have demonstrated that PDL1 was a direct target of the ER BRD4 and the expression inhibition of BRD4 could promote anti‐tumour immunity by modulating PDL1 expression in epithelial ovarian cancer.^35^ TP53 mutation was also found positively correlated to PDL1 expression in LUAD (Figure 3B). Previous results have revealed that the dysfunction of TP53 increased PDL1 expression, helping patients' selection to inhibitors of PDL1 in lung cancer.^36^, ^37^

Given the accordant associations between ER mutations and PD1/PDL1 expression, we next explored if these ER mutations could collectively stratify patient groups with different immune activity. Patients were classified into three subgroups based on the integrated ER mutation status in a cancer type: patients without any ERs mutated (WT group), patients with only one ER mutated (single‐mutation group) and patients with two or more ERs mutated (multiple‐mutation group). By investigating immune characteristics associated with anti‐tumour immune response, we observed obvious differences among the subgroups in multiple cancer types. Compared with WT group, patients with ER mutations showed significantly higher levels of MHC‐I expression, cytotoxic T‐cell infiltration (CTL), M1 macrophage infiltration, CYT and interferon (IFN)‐gamma response in SKCM (Kruskal–Wallis test, *p* < 0.05, Figure 3C, Figure S3A). Accordingly, we observed higher expression of immune effector molecules in patients with the ER mutations (Figure 3D, Figure S3B), such as GZMA and CXCL9. Similar observations were found for LUAD (Figure 3C,D, Figure S3C,D). Patients with the ER mutations had elevated abundances regarding CD8 T and M1 macrophages, and reduced levels regarding immunosuppressive regulatory T cells (Figure S3C). In terms of the immune function related to anti‐tumour immunity,^22^ we found higher T‐cell receptor (TCR) diversity, tumour cell proliferation, wound healing scores and DNA damage measures of nonsilent mutation rate, copy number variation burden and homologous recombination deficiency in the mutation groups of SKCM or LUAD (Figure S4A,B). The deficiencies of DNA damage repair mechanisms can affect immunogenicity of tumours, resulting distinct clinical response of patients to ICB.^38^, ^39^ These findings together indicated that the mutation group for ER mutations involved in PD1–PDL1 dysregulation showed active immune microenvironment with higher immune activity.

### 3.4 Different survival between ER mutation‐based subgroups

**FIGURE 4:**
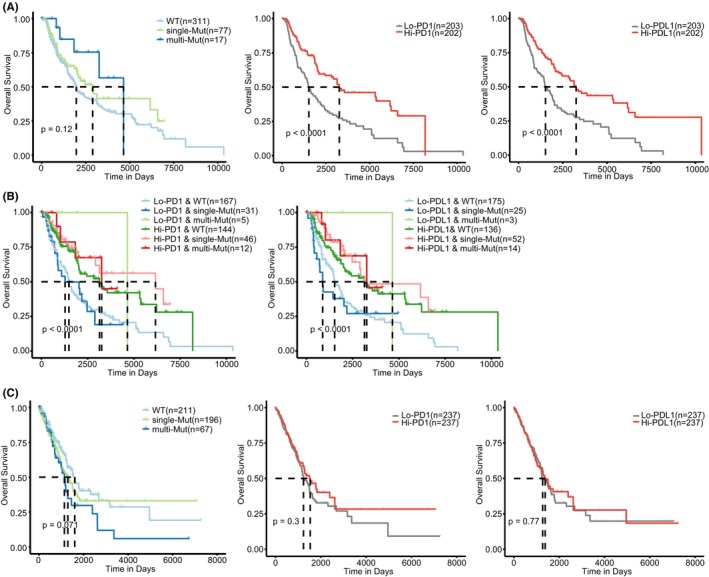
Survival analysis of ER mutations related to PDL1–PD1 interaction in cancer. (A) Survival comparison of patient groups from integrated mutation status of ER mutations (left), expression of PD1 (middle) and PDL1 (right) in SKCM. For ER mutations, the wild type, single‐mutation and multiple‐mutation groups are, respectively, indicated by ‘WT’, ‘single‐Mut’ and ‘multi‐Mut’. For PD1/PDL1 expression, the patients with lower and higher expression (the median value as cut‐off) are respectively indicated by ‘Lo‐PD1/PDL1’ and ‘Hi‐PD1/PDL1’. (B) Kaplan–Meier survival curves among six patient groups stratified by the ER mutation signature and PD1 (left) and PD‐L1 (right). The left and right number of hyphens indicate PD1/PDL1 expression and ER mutation status, respectively. (C) The same as in (A), but for LUAD.

We then investigated the cancer patients' survival associations with the ER mutations involved in PD1–PDL1 dysregulation in TCGA datasets. As expected, we found a higher expression of checkpoints PD1/PDL1 in the mutation groups of SKCM (*p* < 0.001, Figure S5A). Patients with the ER mutations or high checkpoint expression had an improved overall survival (OS) (Figure 4A, Figure S5B). When stratifying melanoma patients by the ER mutations and checkpoint gene expression, we observed a significant survival difference (log‐rank *p* < 0.05, Figure 4B, Figure S5C). Patients in the WT and low checkpoint expression group had a worse OS and progress‐free survival (PFS). In LUAD, patients with the ER mutations also showed higher levels of checkpoint expression (*p* < 0.05, Figure S5D). While, we did not observe a survival difference among combined groups by ER mutations and checkpoint expression (Figure S5E). Patients with the ER mutations showed moderate survival disadvantages (Figure 4C, Table S8), while no survival associations with the checkpoint gene expression were observed in LUAD (Figure 4C, Figure S5F).

### 3.5 Predictive significance of the ER mutation‐based signature for clinical benefit from immunotherapy

**FIGURE 5:**
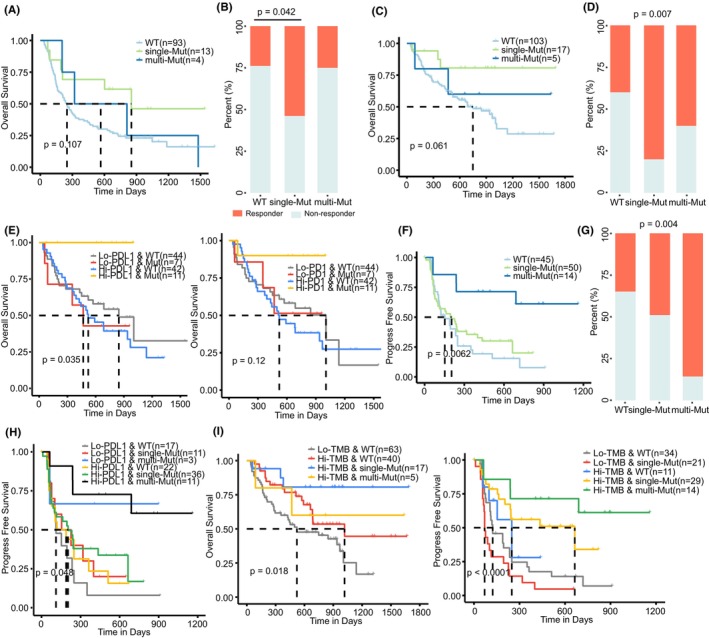
Correlation of ER mutation‐based signature with clinical outcomes of ICB in cancer. (A) Survival comparison for patient groups according to the integrated mutation status of ER mutations for melanoma patients received immune checkpoint blockade **(**ICB) therapy (Van Allen cohort). (B) Rate of clinical response according to the ER mutations related to dysregulated PDL1–PD1 interaction in the Van Allen cohort. Depicted *p* values are from Fisher's exact tests. (C, D) The same as in (A, B), but for melanoma patients of Liu cohort. (E) Kaplan–Meier survival curves among four patient groups stratified by the ER mutation signature and expression of PDL1 (left) and PD1 (right) in the Liu cohort. The left and right number of hyphens indicate PDL1 expression and ER mutation status, respectively. For ER mutations, the wild‐type and mutation groups are, respectively, indicated by ‘WT’ and ‘Mut’. For PD1/PDL1 expression, the patients with lower and higher expression (the median value as cut‐off) are respectively indicated by ‘Lo‐PD1/PDL1’ and ‘Hi‐PD1/PDL1’. (F, G) The same as in (A, B), but regarding lung cancer cohort. (H) Kaplan–Meier survival curves among six patient groups from ER mutation signature and PDL1 expression. The left and right number of hyphens indicate PDL1 expression and ER mutation status, respectively. (I) Kaplan–Meier survival curves among patient groups from ER mutation signature and TMB in the Liu (left) and lung cancer (right) cohorts. The left and right number of hyphens indicate TMB levels and ER mutation status, respectively.

Cancer patients with inhibitory immune checkpoints PD1 and PDL1 expression were reported to have a better clinical response to ICB.^40^, ^41^, ^42^ Therefore, we investigated if the ER mutations involved in the PD1–PDL1 dysregulation could predict patients' response to immunotherapy. The Tumour Immune Dysfunction and Exclusion (TIDE) score was previously shown to have a good performance in melanoma patients' response prediction to ICB.^43^ We calculated the TIDE scores in TCGA SKCM samples and observed lower scores in the multiple‐mutation group (Figure S6A). Accordingly, a higher proportion of these patients were predicted to response to ICB (Fisher's exact test, *p* = 0.045 for the multiple and WT groups). Using the genomic profiles of melanoma samples treated with anti‐CTLA4 (Van Allen cohort),^44^ we classified these samples into three groups according to the mutation signature obtained by TCGA data. Patients in the mutation group had a longer OS (Figure 5A, log‐rank *p* = 0.107; hazard ratio [HR]_single vs. WT_ = 0.421, 95% CI = 0.183–0.969, *p* = 0.042). Accordingly, patients with ER mutations had a higher response (Figure 5B, Fisher's exact test, *p* = 0.042 for the single and WT groups). For an independent dataset regarding melanoma samples treated with anti‐PD1 (Liu cohort),^45^ the mutation group showed an improved OS relative to the WT group (Figure 5C, log‐rank *p* = 0.061; HR_single vs. WT_ = 0.277, 95% CI = 0.087–0.887, *p* = 0.031). Compared to the WT group, higher response rates were also observed in the mutation group (Figure 5D, Fisher's exact test, *p* = 0.005 for the single and WT groups). Especially, we found significant survival differences among the combined groups stratified by the ER mutations and checkpoints expression. Patients with the ER mutations and high checkpoints had an improved OS (Figure 5E, *p* = 0.035 for PDL1 and *p* = 0.12 for PD1). For patients with high PDL1 expression, we also observed a significant survival advantage in the mutation group (Figure 5E, log‐rank *p* = 0.020). These observations were also found in an integrated lung cancer cohort^46^, ^47^ (Figure 5F–H). Patients in the multiple‐mutation group had a significantly better PFS (log‐rank *p* = 0.006, Figure 5F) and clinical response to ICB (Figure 5G, Fisher's exact test, *p* = 0.004). Furthermore, focused on the patients group with high PDL1 levels, the multiple‐mutation group still had an improved survival (Figure 5H).

Tumour mutation burden (TMB) is typically a positive indicator of ICB outcome,^48^ we then stratified patients with low/high TMB by the ER mutations and compared the survival differences. As expected, we observed a significantly higher TMB in the patients with mutations in one or more ER genes (*p* < 0.05, Figure S6B). The univariate analysis indicated that the ER mutations showed more positive associations with patients' survival relative to TMB (lower HR values, Tables S9 and S10). In both cohorts of Liu and lung cancer, patients in the ER mutation and TMB high group had significant survival advantages (log‐rank *p* < 0.05, Figure 5I). Notably, we still found survival differences between the ER mutation and WT groups for the patients with high TMB. These observations together demonstrated that the ER mutations involved in the dysregulated PD1–PDL1 interaction may be useful in predicting response to ICB and improving cancer patients' stratification.

## 4 DISCUSSION

ERs are essential for cancer development through the modulation of gene expression. Dysfunction of ERs can disturb anti‐tumour immune response, causing a suppressive immune microenvironment. Despite this, there remains a significant gap in knowledge regarding the impact of ER dysfunction on ligand–receptor interaction perturbations in the tumour microenvironment. Here, we developed a dedicated method, MERIN, to decode the functional consequences of ER mutations on molecular interactions in cancer by integrating gene expression, somatic mutation and molecular interaction data. Using MERIN, we comprehensively uncovered functional consequences of ER mutations in the context of dysregulated ligand–receptor interactions across 33 cancer types. Particularly, we revealed the potential clinical relevance of ER mutations in cancer patients' stratification. Different patient groups showed distinct immune profiles, survival and ICB therapy response.

The dysfunction of ERs caused by their genetic alterations or expression changes can induce epigenetic alterations of downstream genes, resulting in cancer development and poor survival.^13^, ^25^ Ligand–receptor pairs mediating the complicated and dynamic communication between cells underline the molecular basis of tumour immune response. Emerging researches reported that the ERs with expression dysregulation could modulate the immune microenvironment.^49^, ^50^ However, there is a significant gap in understanding the epigenetic determinants triggering changes of the immune microenvironment. Li et al. recently proposed a network‐based method to identify candidate genomic mutations that perturb functional pathways, however, they focused on the general PPI network or human signalling pathways, and only identified mutations occurred in the interacting genes themselves.^26^ Inspired by Li et al., we designed MERIN which can systematically identify ER mutations involved in dysregulated molecular interactions in the immune microenvironment. This study represents the initial effort to thoroughly illustrate the functional impact of ER mutations on 3182 immune‐related ligand–receptor interactions. As a classical tumour suppressor gene, TP53 mutations were found to be associated with these dysregulated interactions in 26 cancer types (Figure S1A). Indeed, the mutations of TP53 can cause immune escape through affecting the expression of immune checkpoints or recruitment of immune cells.^15^ Moreover, we observed the associations between KMT2 family mutations and the dysregulated interactions in 19 cancer types (Figure S1A). The genomic mutations in the KMT2 family could predict the better clinical response of patients to ICB therapy in multiple cancers.^16^ Functional enrichment analysis revealed that the dysregulated ligand–receptor genes mediated by ER mutations were enriched in immune‐related function terms (Figure 2A). The gene modules identified from the PPI network further demonstrated the vital functional roles of ER mutations in tumour immunity (Figure 2B). The gene modules containing TP53 and JAK2 were annotated with leukocyte proliferation and regulation of adaptive immune response, respectively, suggesting their roles in tumour immune response.

ICB therapy targeting the PD1–PDL1 interaction has been successfully applied in multiple cancers, however, the limited response highlights the intrinsic behaviour of tumour immune evasion. In fact, we found ER mutations that were involved in the dysregulation of PD1–PDL1 interaction in different cancers (Figure 3A). For melanoma and lung cancers, we observed upregulation of PD1 and PDL1 in the ER mutation samples (Table S7). Previous studies reported that dysfunction of TP53 could increase the expression of PDL1, thereby suppressing T cell activity.^15^ As a direct target of BRD4, PDL1 expression could be suppressed by the dysfunction of BRD4 in epithelial ovarian cancer. This suppression increased the anti‐tumour immunity.^35^ When stratifying patients by integrated mutational status of ER mutations involved in the PD1–PDL1 interaction, we observed different immune microenvironment among the patient groups (Figure 3C,D, Figures S3 and S4). Tumours in the mutation group showed an active immune microenvironment with higher immune activity.

Moreover, we found a positive association between the ER mutations and immunotherapy response. Based on the genomic profiles for ICB‐treated patients, we observed that the mutation group in different cancers had both better survival and higher clinical response rates (Figure 5A–D,F,G). Immune checkpoints expression and TMB usually indicate better clinical outcomes of ICB, but they had limitations.^51^ Combining the ER mutations and PD1/PDL1 expression, we observed improved survival for patients in the ER mutation and checkpoint high group (Figure 5E,H). The survival difference was also found for the combination with TMB, demonstrating the improved patient stratification when considering ER mutations (Figure 5I). Notably, a higher TMB was observed in the mutation group (Figure S6B). Importantly, epigenetic drugs could enhance ICB efficacy in cancer treatment^52^, ^53^ and significant breakthroughs have been made in overcoming immunotherapy resistance by modulating immune response.^14^ For example, the pharmacological inhibition of the ER HDAC1 could restore anti‐tumour immune response^17^ and potentiate ICB efficacy through upregulating PDL1 expression.^18^, ^19^ Altogether, we revealed the potential targets of epi‐drugs that can be used coordinately with cancer immunotherapy, thereby strengthening the therapy efficacy.

This study, although providing valuable biological insights into the functional implications of ER mutations in the context of immune‐related ligand–receptor interactions, is subject to certain limitations. First, our application of MERIN for identifying the associations between ER mutations and ligand–receptor interactions needs to be validated by experimental data. Second, a more precise understanding of the molecular mechanism underlying dysregulated immune microenvironment can be gained by integrating information from single‐cell sequencing data. Third, animal experiments are necessary to dissect the functional roles of ER mutations in the immune microenvironment. Given the intricate relationship among tumour genomics, epigenomics and anti‐tumour immune responses, we anticipate a better prediction performance of cancer immunotherapy can be obtained through incorporating our biomarkers with additional information on microenvironment cells such as spatial distribution.

We proposed a novel approach, MERIN, to decode the epigenetic impact on perturbed ligand–receptor interactions by harnessing multi‐omics information, leading to a systematical exploration of novel insights into the molecular mechanisms underlying the tumour immune microenvironment. The identified dysfunctional ERs also showed significant clinical implications in cancer patients' survival and immunotherapy response. Our study highlights the potential of MERIN in pinpointing ER mutations deserved to be further investigated, and we anticipate the application of MERIN will facilitate the development and refinement of immunotherapeutic strategies.

## AUTHOR CONTRIBUTIONS

**Aiai Shi:** Conceptualization (equal); data curation (equal); formal analysis (lead); methodology (equal); project administration (equal); visualization (lead); writing – original draft (lead); writing – review and editing (equal). **Chaohuan Lin:** Data curation (equal); writing – review and editing (supporting). **Jie Lyu:** Conceptualization (equal); funding acquisition (lead); methodology (equal); project administration (equal); writing – review and editing (equal).

## FUNDING INFORMATION

This work was supported by the National Natural Science Foundation of China [grant number 32170665 to J.L.] and Wenzhou Institute, University of Chinese Academy of Sciences' startup fund [grant number WIUCASQD2021006 to J.L.].

## CONFLICT OF INTEREST STATEMENT

The authors declare no conflicts of interest.

## Supporting information


Data S1.

Table S1.
Table S2.
**Table S3.**.
Table S4.
Table S5.
Table S6.
Table S7.

## Data Availability

The data for analyses in this study are available in the article or from the authors upon reasonable request. We provided the codes of MERIN at https://github.com/syy2017/MERIN.
