# Supplementary material for: Decoding functional impact of epigenetic regulator mutations on ligand–receptor interaction perturbations for evaluation of cancer immunotherapy
Source: J Cell Mol Med. 2024 Sep 25;28(18):e70009. doi: 10.1111/jcmm.70009 (PMC11424496; doi:10.1111/jcmm.70009)
Supplement: Supplementary file 1 — Data S1. [file JCMM-28-e70009-s001.docx]

**Supplemental Figures**


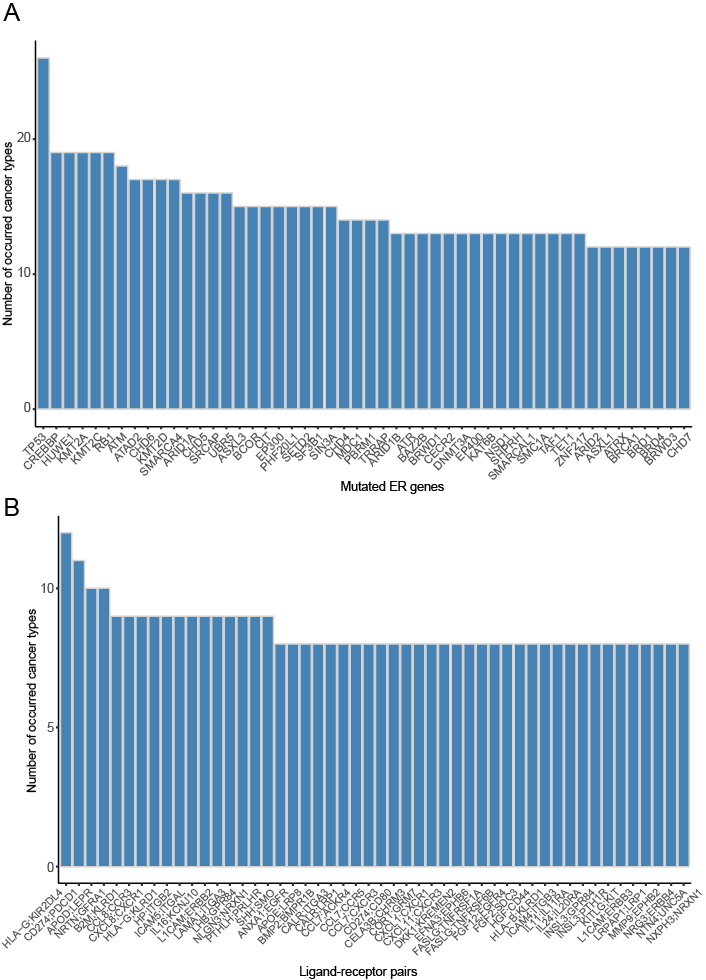


**Figure S1. The distribution of ER mutations involved in dysregulated molecular interactions across different cancer types.** (A) Bar plots showing the number of occurred cancer types for top 50 mutated ER genes across 33 cancers. (B) Bar plots showing the number of occurred cancer types for top 50 dysregulated ligand-receptor interaction pairs across 33 cancers.


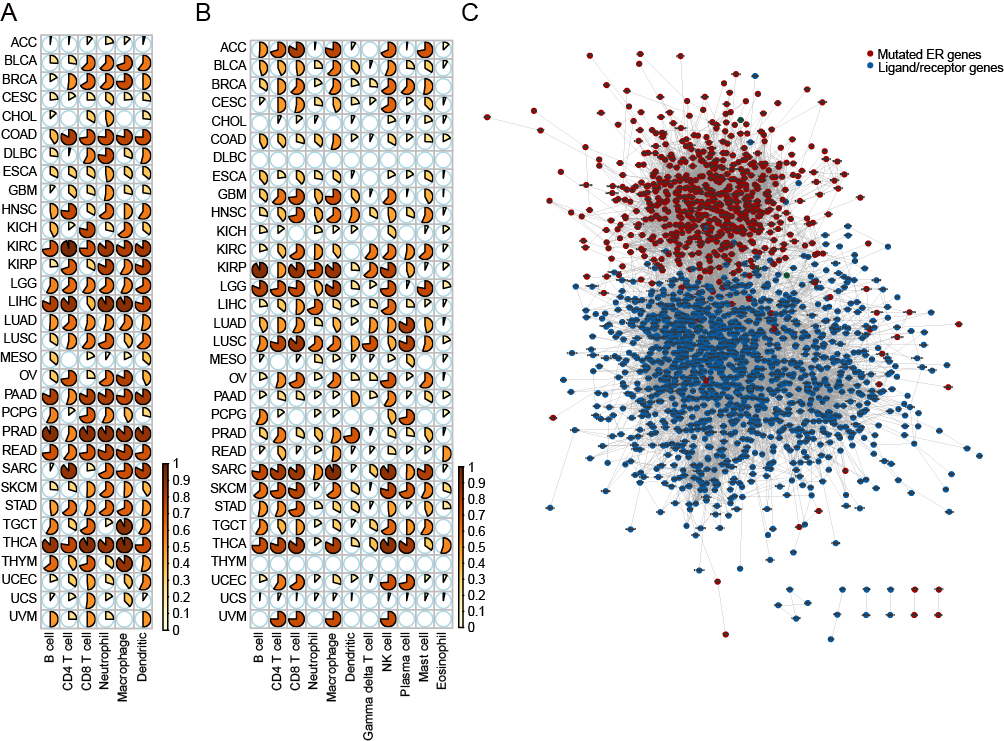


**Figure S2. Functional characterization of identified ERs across cancer types.** (A) The proportion of identified ERs related to immune cell infiltration estimated by TIMER in each cancer. The ERs expression and immune infiltrates with Spearman's correlations > 0.1 and p values < 0.05 are selected. (B) The proportion of identified ERs related to immune cell infiltration estimated by CIBERSORT in each cancer.  (C) The subnetwork of protein interactions showing ER mutations mediated perturbed ligand-receptor pairs across 33 cancer types. Red, mutated ER genes; blue, ligand/receptor genes.


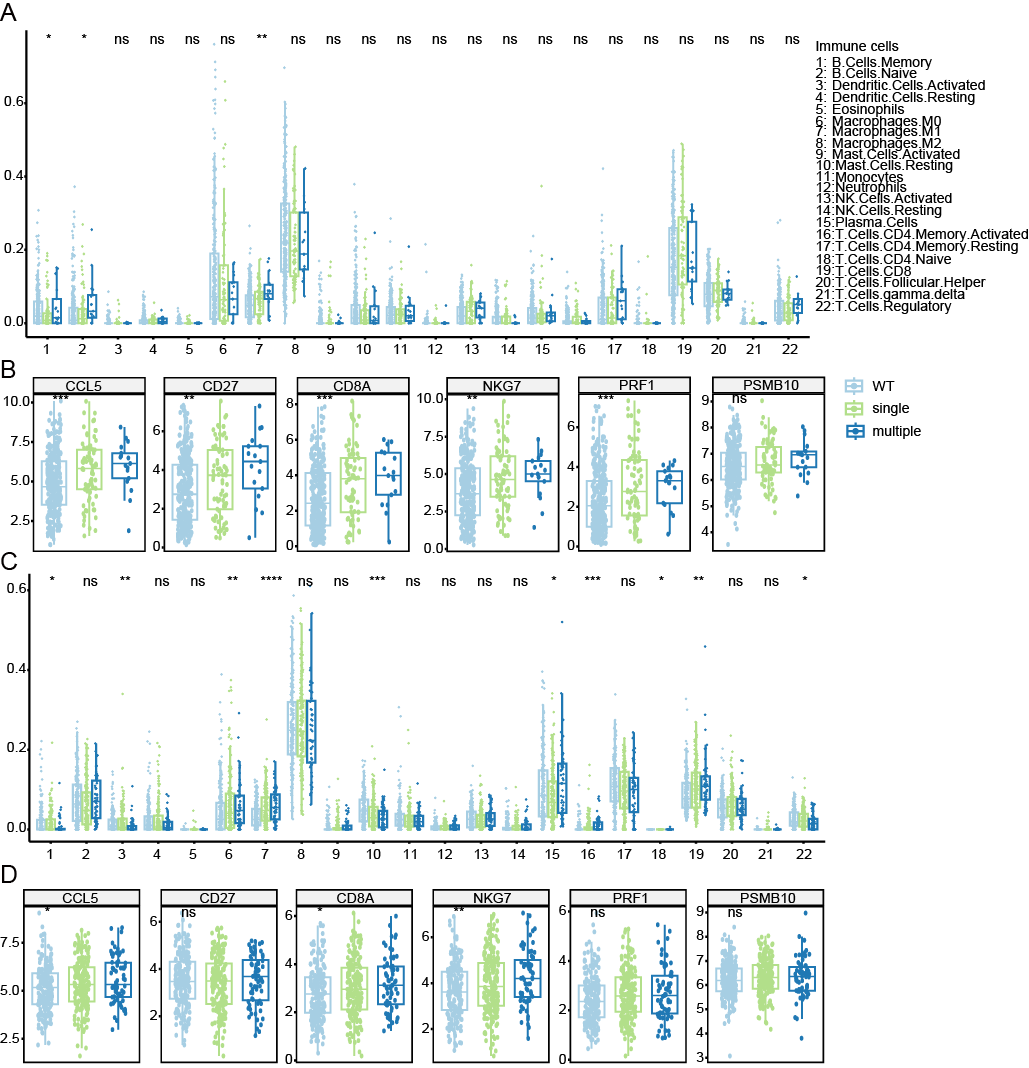


**Figure S3. The immune microenvironment characterization of different patient groups.** Boxplots showing the distribution of infiltration levels of immune cell types estimated by CIBERSORT in three mutation groups for SKCM (A) and LUAD (C). Comparison for the expression of six immune effector molecules in three mutation groups for SKCM (B) and LUAD (D). ns, non-significant, * p < 0.05, ** p < 0.01, *** p < 0.001, and **** p < 0.0001.


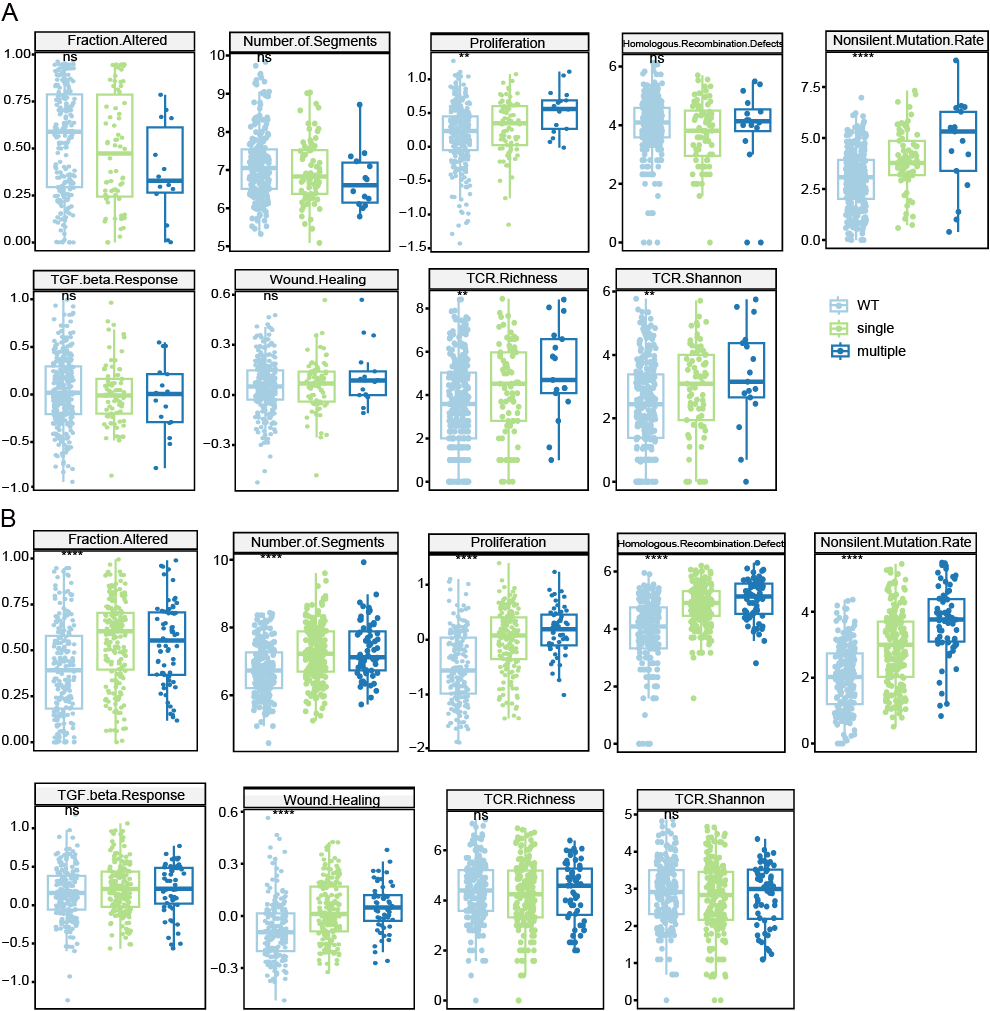


**Figure S4. Association between the ER mutation signature and immune characteristics.** Comparison of copy number variation (CNV) burden (fraction of genome alterations and number of segments), tumor cell proliferation, HRD score, nonsilent mutation rate, transforming growth factor (TGF)-beta response, wound healing scores and T cell receptor (TCR) diversity (richness and shannon) in three mutation groups for SKCM (A) and LUAD (B). ns, non-significant, * p < 0.05, ** p < 0.01, *** p < 0.001, and **** p < 0.0001.


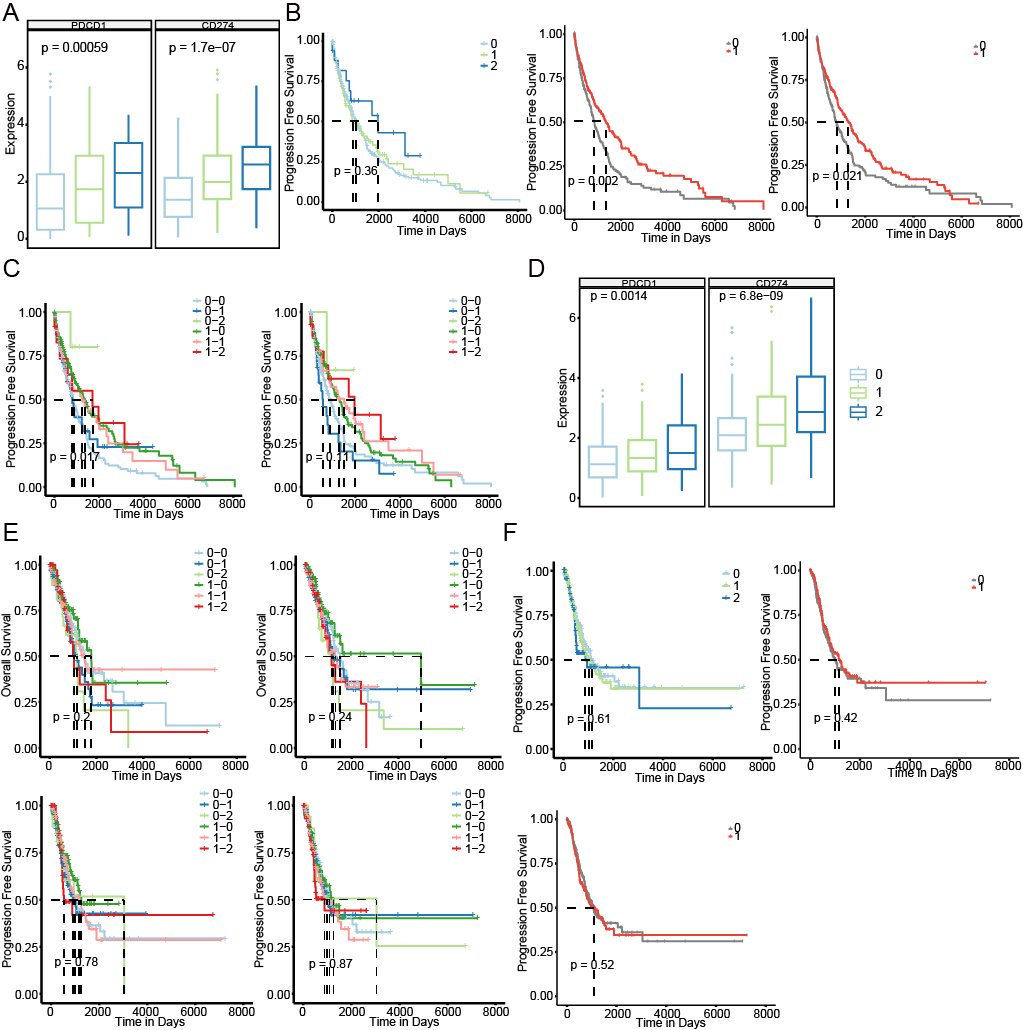


**Figure S5. Prognostic associations of the ER mutation signature in cancer.** (A) The expression distribution of PD1 (left) and PDL1 (right) among the three mutation groups in SKCM. (B) Survival comparison of patient groups according to the integrated mutation status of ER mutations (left), the expression of PD1 (middle) and PDL1 (right) in SKCM. For ER mutations, the wild type, 'single mutant' and 'multiple mutant' groups are respectively indicated by '0', '1' and '2'. For PD1/PDL1 expression, the patients with lower and higher expression (the median value as cut-off) are respectively indicated by '0' and '1'. (C) Kaplan-Meier survival curves among six patient groups stratified by the ER mutation signature and expression of PD1 (left) and PD-L1 (right). The left and right number of hyphens indicate PD1/PDL1 expression and ER mutation status, respectively. (D-F) The same as in (A-C), but for LUAD.


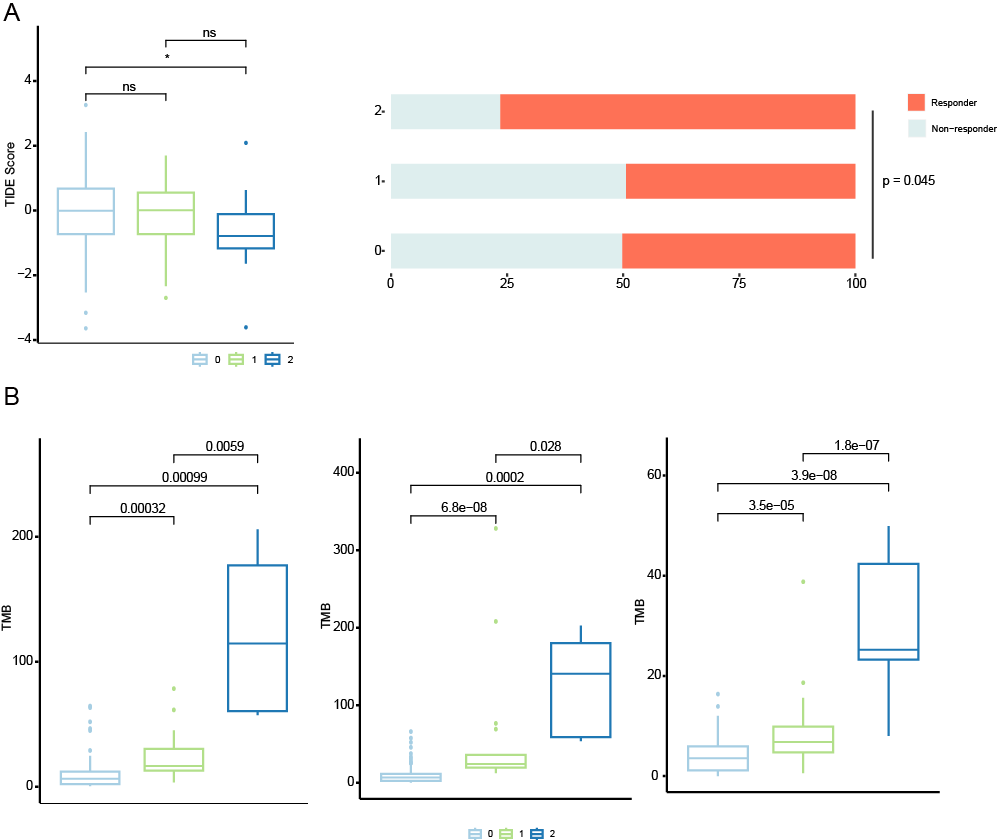


**Figure S6. Analysis of association between ER mutations involved in PDL1-PD1 interaction and clinical response of ICB in cancer.** (A) The difference of TIDE score and rate of clinical response predicted by TIDE among ER mutation groups in TCGA-SKCM cohort. For ER mutations, the wild type, 'single mutant' and 'multiple mutant' groups are respectively indicated by '0', '1' and '2'. (B) The distribution of TMB among three ER mutation groups in Van Allen (left), Liu (middle) and lung cancer (right) cohorts.

## Supplemental Tables

**Table S1. The cancer types used in our study.**

**Table S2. List of human Epigenetic regulators (ERs) used in our analysis.**

**Table S3. The ligand-receptor pairs for our study.**

**Table S4. The top ER-interaction pairs across cancer types.**

**Table S5. The proportions of interaction-related ERs associated with immune cell infiltration from TIMER and CIBERSORT.**

**Table S6. The top biological function for subnetwork modules identified by MCODE.**

**Table S7. The log2FC of dysregulated immune interaction pairs between mutant and wild type samples.**

**Table S8. Uni- and multivariate analysis for overall survival in cancer.**

| Variable |  | Univariate | | | Multivariate | | |
| --- | --- | --- | --- | --- | --- | --- | --- |
|  |  | HR | 95%CI | P value | HR | 95%CI | P value |
| **LUAD** | |  |  |  |  |  |  |
| Mutation signature | | | | | | | |
| single_Mut VS WT 1.287 0.928-1.783 0.1303 1.211 0.867-1.692 0.2608  multi_Mut VS WT 1.600 1.045-2.451 **0.0305** 1.674 1.090-2.571 **0.0186** | | | | | | | |
| Age 1.008 0.993-1.024 0.3025 1.012 0.997-1.028 0.1251 | | | | | | | |
| Gender (male VS female) | | 1.099 | 0.816-1.480 | 0.5351 | 1.047 | 0.776-1.413 | 0.7631 |
| Stage ( III/IV VS 0/I/II) | | 2.476 | 1.811-3.386 | **<0.0001** | 2.466 | 1.798-3.384 | **<0.0001** |

^a.^CI, confidence interval; HR, hazard ratio. The bold values refer to *P* < 0.05 indicated significant statistical difference.

**Table S9. Uni- and multivariate analysis for progress-free survival in each data set of non-small cell lung cancer.**

| Variable |  | Univariate | | | Multivariate | | |
| --- | --- | --- | --- | --- | --- | --- | --- |
|  |  | HR | 95%CI | P value | HR | 95%CI | P value |
| **Lung cancer cohort ( Hellmann + Rizvi, N = 109 )** | |  |  |  |  |  |  |
| Mutation signature | |  |  |  |  |  |  |
| single_Mut VS WT | | 0.801 | 0.495-1.296 | 0.366 | 0.960 | 0.571-1.612 | 0.8764 |
| multi_Mut VS WT | | 0.232 | 0.089-0.604 | **0.0028** | 0.647 | 0.182-2.310 | 0.5029 |
| Age | | 0.979 | 0.955-1.004 | 0.1051 | 0.962 | 0.936-0.989 | **0.0064** |
| Gender (male VS female) | | 1.212 | 0.762-1.928 | 0.4179 | 1.228 | 0.756-1.996 | 0.4072 |
| TMB | | 0.942 | 0.911-0.974 | **5e-04** | 0.940 | 0.895-0.987 | **0.0137** |
| **Hellmann cohort (N = 75)** | |  |  |  |  |  |  |
| Mutation signature | |  |  |  |  |  |  |
| single_Mut VS WT | | 1.062 | 0.588-1.916 | 0.8424 | 1.188 | 0.619-2.280 | 0.6055 |
| multi_Mut VS WT | | 0.219 | 0.073-0.655 | **0.0066** | 0.413 | 0.078-2.189 | 0.2984 |
| Age | | 0.985 | 0.958-1.013 | 0.2918 | 0.967 | 0.937-0.998 | **0.0386** |
| Gender (male VS female) | | 1.029 | 0.590-1.794 | 0.9211 | 1.122 | 0.613-2.054 | 0.7101 |
| TMB | | 0.952 | 0.921-0.985 | **0.0045** | 0.964 | 0.909-1.022 | 0.217 |
| **Rizvi cohort (N = 34)** | |  |  |  |  |  |  |
| Mutation signature | |  |  |  |  |  |  |
| Mut VS WT | | 0.423 | 0.175-1.024 | **0.0564** | 0.611 | 0.238-1.572 | 0.3069 |
| Age | | 0.956 | 0.898-1.018 | 0.1595 | 0.954 | 0.904-1.007 | **0.0888** |
| Gender (male VS female) | | 1.790 | 0.768-4.174 | 0.1775 | 1.678 | 0.687-4.101 | 0.2562 |
| TMB | | 0.869 | 0.784-0.964 | **0.0078** | 0.873 | 0.778-0.979 | **0.0203** |

^a.^CI, confidence interval; HR, hazard ratio. The bold values referred to *P* < 0.1. The two samples in ‘multi_Mut’ were grouped into the ‘single_Mut’ as the ‘Mutant’ group.

**Table S10. Uni- and multivariate analysis for overall survival in each data set of melanoma.**

| Variable |  | Univariate | | | Multivariate | | |
| --- | --- | --- | --- | --- | --- | --- | --- |
|  |  | HR | 95%CI | P value | HR | 95%CI | P value |
| **Van Allen cohort (N = 110)** | |  |  |  |  |  |  |
| Mutation signature | |  |  |  |  |  |  |
| single_Mut VS WT | | 0.421 | 0.183-0.969 | **0.0419** | 0.485 | 0.206-1.141 | **0.0974** |
| multi_Mut VS WT | | 0.808 | 0.293-2.229 | 0.681 | 1.444 | 0.326-6.391 | 0.6285 |
| Age | | 1.004 | 0.991-1.018 | 0.5475 | 1.007 | 0.993-1.021 | 0.358 |
| Gender (male VS female) | | 0.779 | 0.489-1.240 | 0.2928 | 0.731 | 0.445-1.200 | 0.2154 |
| TMB | | 0.995 | 0.987-1.003 | 0.2184 | 0.993 | 0.982-1.005 | 0.2419 |
| **Liu cohort (N = 125)** | |  |  |  |  |  |  |
| Mutation signature | |  |  |  |  |  |  |
| single_Mut VS WT | | 0.277 | 0.087-0.887 | **0.0306** | 0.302 | 0.084-1.088 | **0.0671** |
| multi_Mut VS WT | | 0.648 | 0.158-2.661 | 0.5473 | 0.709 | 0.135-3.732 | 0.6852 |
| Gender (male VS female) | | 0.803 | 0.484-1.332 | 0.3949 | 0.835 | 0.502-1.389 | 0.4874 |
| TMB | | 0.994 | 0.985-1.003 | 0.2029 | 0.999 | 0.989-1.009 | 0.8167 |

^a.^CI, confidence interval; HR, hazard ratio. The bold values referred to *P* < 0.1.
